# Comparative Analysis of Artificial Intelligence-Generated and Human-Written Personal Statements in Emergency Medicine Applications

**DOI:** 10.7759/cureus.88818

**Published:** 2025-07-26

**Authors:** Elizabeth Steele, Anthony Steratore, Brian Z Dilcher, Kathryn Bandi

**Affiliations:** 1 Emergency Medicine, West Virginia University School of Medicine, Morgantown, USA; 2 Emergency Medicine, University of Pittsburgh Medical Center, Pittsburgh, USA

**Keywords:** artificial intelligence, medical education, personal statements, residency application, student advising

## Abstract

Introduction

Personal statements (PSs) have long been part of the Electronic Residency Application Service (ERAS) application; however, there exist only limited guidelines for their creation and even fewer for their role in the application review process. Applicants invest significant time in writing their PSs, and still, program directors rank PSs among the least important factors in interview and rank order list decisions. The emergence of generative artificial intelligence (AI), particularly large language models (LLMs) like ChatGPT, has introduced questions of ethics and originality across all aspects of education, particularly in the generation of free-form documents such as the PS. This study evaluates whether AI-generated PSs are distinguishable from authentic ones written by applicants and their perceived impact on residency selection.

Methods

Five AI-generated PSs were created using ChatGPT, incorporating applicant location, hobbies, and career background. Five de-identified, authentic PSs randomly selected from incoming emergency medicine (EM) interns were used for comparison. A Qualtrics survey was distributed electronically to the Council of Residency Directors (CORD) community. Respondents rated the PSs on writing quality, ability to convey personal attributes, and perceived influence on interview decisions. Statistical analyses (ANOVA and Wilcoxon tests) were used to assess differences between AI-generated and authentic statements.

Results

A total of 66 responses were collected over a two-month period. Of these, eight respondents did not regularly review ERAS applications, and 28 did not complete the survey beyond the initial question, leaving 30 responses for analysis. There were no statistically significant differences between AI-generated and authentic PSs in grammar and writing style (p = 0.5897), expression of personal attributes (p = 0.6827), overall quality (p = 0.2757), or perceived influence on interview decisions (p = 0.5457). Free-text comments reflected skepticism about the value of the PS in the selection process.

Conclusion

AI-generated PSs performed comparably to authentic ones, potentially further challenging the relevance of PSs in residency applications. These findings suggest an inherent lack of originality in the PS and may support re-evaluating the role of the PS and even exploring more meaningful ways to assess applicant fit in the residency selection process. Novel methods, such as structured interviews, standardized video responses, personality inventories, or situational judgment tests, may be considered to supplement the role intended for the PS.

## Introduction

Personal statements (PSs) have long been a part of the application process, challenging students with writing a lengthy narrative that describes their personal attributes and interests to prospective program directors. Despite applicants' perception of its importance, the Electronic Residency Application Service (ERAS) provides minimal guidance, beyond the 28,000-character limit, about the content or even purpose of this document [1]. Data on applicants' feelings about PSs is scarce, especially in the field of emergency medicine (EM), as are studies reporting the time investment by students and their advisors in these statements. Orthopedic applicants spent an average of 15 hours on their PSs, a considerable time investment in a busy schedule often occupied by audition rotations [2]. The majority of those surveyed also reported using either a physician mentor or advisor to edit these statements [2]. It is an often-acknowledged role of a student advisor or clerkship director to assist students with this process, thus a considerable and unremunerated time investment for them as well.

Fourth-year medical students have reported difficulties assessing their fit in a program with virtual interviews [3]. Many programs are similarly challenged by the limitations of virtual interviews and are working to find better methods to assess the personal qualities of applicants and their fit within the programs [3]. Despite this growing need, the PS remains limited in its perceived utility. Data on the role of the PS in EM applications is scarce; however, there is evidence of poor inter-rater reliability and significant heterogeneity in their content and perceived purpose [4]. The further possibility that this poor inter-rater reliability may reflect applicant reviewer biases puts PSs at cross-purposes with ongoing endeavors to remove bias from the residency application process. Unsurprisingly, EM program directors rank PSs in the bottom third of importance of personal characteristics when deciding which applicants to invite to interviews and as an even less important factor in constructing a rank list [5].

With the rapid proliferation of generative artificial intelligence (AI), especially large language models (LLMs) such as ChatGPT, one can generate a de novo document in a matter of seconds. LLMs are generative pre-trained transformers (GPTs), which are trained on massive amounts of data and use neural networks to process natural language from the prompt, predicting the probabilities of the next word in a response based on the context of the prompt and the previous words generated. Requiring only loosely associated content ideas, formatting instructions, and length guidelines, a prompt can easily be created and submitted to an LLM, which can generate a robust output that satisfies the parameters set forth. The applications of this technology are limitless and have already had an obvious and profound impact on healthcare and education [6,7].

Anecdotally, many of our students have already reported using ChatGPT or similar tools to edit assignments, including their PSs. This inspired us to experiment with just how well ChatGPT could develop an entire PS. After minimal prompting and exploration, it became evident, at least to us, that ChatGPT could write PSs that passed as authentic. We questioned whether these PSs would appear authentic to others who regularly evaluate them as part of the evaluation process, theorizing that if they did, it would at least imply a trend towards inherently unoriginal and thus impersonal statements in general. Previous studies have shown that application evaluators cannot distinguish between AI-generated PSs and those created by humans [8,9]. In this study, it is hypothesized that AI-generated PSs will be evaluated similarly in terms of quality to authentic PSs in the eyes of EM application evaluators and that these evaluators will demonstrate significant heterogeneity in their opinions on the utility of PSs.

## Materials and methods

This study was acknowledged as exempt by the West Virginia University Institutional Review Board (protocol number: 2308830215) prior to the collection of any data.

Five profiles of potential applicants were created using the following extremely simple prompt to be used to instruct ChatGPT 3.5 to write the PS: "Write a one-page personal statement essay for a medical student applying for an emergency medicine residency program. The applicant is from (location), has an interest in (hobbies), and they were previously a (career experience)."

ChatGPT, via a standard internet browser, used only the above prompt combined with five different combinations of location, hobbies, and career experience. ChatGPT then generated five PSs. These statements took less than a minute each to generate, and we used the first output drafts. Our incoming PGY-1 interns were surveyed, and all 10 voluntarily submitted their PS from their residency application. We then randomly selected five of their PSs for use in the survey. Two separate researchers verified that pieces of potentially identifying information were redacted, these being almost solely references to locations. No other content edits of any kind were made in either cohort.

The 10 PSs were then added to a Qualtrics survey along with seven questions, including one optional free-text question, intended to assess the quality of the PSs and their effect on a potential interview invitation in a hypothetical application (Qualtrics, Provo, Utah). The Qualtrics software was programmed to use its randomization function to assign three PSs, a number determined to reduce fatigue and improve response rate in completing the evaluation, as well as corresponding questions to each respondent. The survey was distributed by posting it to the Council of Residency Directors (CORD) Survey community, which has over 2,500 registered community members. Prospective participants were only provided with the prompt below, the survey, and the IRB cover letter.

"My colleague and I are conducting research on the utility of the PS in EM applications. If you are involved in evaluating ERAS applications and could spare a few minutes to read and evaluate a few example PSs, we would deeply appreciate it. The link is just below, and we have also attached a QR code for your convenience. Your participation is completely anonymous, and all findings will be reported in an aggregate. We greatly appreciate any help!"

This prompt was the only instruction provided to the respondents, and no other information regarding the PSs, nor any mention of AI, was provided, effectively blinding them to the nature of the three randomized PSs they would be assigned to review. A total of 66 responses were collected from August 2022 to September 2022. The post was repeated once as a reminder toward the end of the data collection period.

Raw data were downloaded from Qualtrics to a Microsoft Excel (Microsoft Corporation, Redmond, Washington) file and then analyzed using descriptive statistics with SAS 9.4 (SAS Institute Inc., Cary, NC, USA) [10]. ANOVA was used to assess whether there was a statistically significant difference in the average rating between the ChatGPT and human groups for writing quality, personal attributes, and comparison of the PS with other statements (Q1 to Q3). Due to the non-normality of the ratings, the Wilcoxon rank sum test was also calculated to assess the reliability of the ANOVA significance test. The chi-square test of independence was calculated to assess whether there was a significant difference between the groups in whether the PS was used in interview decisions (Q4), had an adverse impact on interview decisions (Q5), or whether there was a need for clarifying questions (Q6). An alpha of 0.05 was selected as the threshold for statistical significance.

## Results

We received a total of 66 responses to the survey. Eight responses of the total 66 (12.1%) were screened out due to answering "no" to the initial question that verified they consistently reviewed residency applications in their duties, leaving us with 58 respondents in our intended demographic. Of these remaining, 26/58 (39.4%) were considered complete, having evaluated all three PSs. We had 28/58 (48.3%) responses that did not proceed further than the initial question and 4/58 (6.9%) that answered questions on at least one PS but did not complete the entirety of the survey for subsequent PSs.

After initial verification that they consistently reviewed residency applications as part of their duties, the respondents assessed various dimensions, ranking them in categorical tiers, including grammar/writing style, personal attributes, and the overall quality of the statements. Additionally, the respondents reported their perceived utility of PSs in the interview selection process and the necessity for follow-up questions. The results are summarized in Table 1 below.

**Table 1:** Aggregated results of the survey responses comparing ChatGPT-generated personal statements to authentic personal statements.

Question	ChatGPT	Authentic	P-value (ANOVA)	P-value (Wilcoxon)	P-value (Chi-square)
Letter Origin	38 (46.3%)	44 (53.7%)	-	-	-
Q1: Grammar/Writing Style					
Upper One-third	19 (26.3%)	13 (29.6%)	0.5897	0.6109	-
Middle One-third	24 (63.2%)	28 (63.6%)	-	-	-
Lower One-third	4 (10.5%)	3 (6.8%)	-	-	-
Q2: Conveying Personal Attributes					
Upper One-third	11 (29.0%)	10 (22.7%)	0.6827	0.7541	-
Middle One-third	19 (50.0%)	30 (68.2%)	-	-	-
Lower One-third	8 (21.1%)	4 (9.1%)	-	-	-
Q3: Comparison Across Cycles					
Upper One-third	6 (15.8%)	10 (22.7%)	0.2757	0.2788	-
Middle One-third	26 (68.4%)	30 (68.2%)	-	-	-
Lower One-third	6 (15.8%)	4 (9.1%)	-	-	-
Q4: Use in Interview Decisions					
Yes	29 (76.3%)	36 (81.8%)	-	-	0.5399
No	9 (23.7%)	8 (18.2%)	-	-	-
Q5: Adverse Impact on Interview Decision					
Yes	5 (13.2%)	1 (2.3%)	-	-	0.0591
No	33 (86.6%)	43 (97.7%)	-	-	-
Q6: Need for Clarifying Questions					
Yes	10 (26.3%)	10 (22.7%)	-	-	0.7059
No	28 (73.7%)	34 (77.3%)	-	-	-

To evaluate grammar and writing style in PSs, the respondents evaluated the PSs using ChatGPT and categorized them into three quality tiers: upper one-third, middle one-third, and lower one-third. In the upper one-third, 26.3% of ChatGPT-generated PSs and 29.6% of authentic PSs were rated in this category. In the middle one-third, 63.2% of ChatGPT PSs and 63.6% of applicant PSs received ratings in this category. In the lower one-third, 10.5% of ChatGPT PSs and 6.8% of authentic PSs were rated. ANOVA and Wilcoxon p-values (0.5897 and 0.6109, respectively) indicated no statistically significant difference between the groups.

The ability to evaluate the extent to which PSs conveyed unique personal attributes was similarly evaluated. Only 29.0% of ChatGPT-generated PSs and 22.7% of authentic PSs were rated in the upper one-third. A total of 50.0% of ChatGPT and 68.2% of applicant PSs were rated in the middle one-third, while the lower one-third included 21.1% of ChatGPT and 9.1% of authentic PSs. ANOVA and Wilcoxon p-values (0.6827 and 0.7541, respectively) indicate no statistically significant difference between ChatGPT and applicant-generated PSs in this area.

When asked how PSs compared to others reviewed across application cycles, 15.8% of ChatGPT-generated PSs and 22.7% of authentic PSs were rated in the upper one-third. The middle one-third included 68.4% of ChatGPT and 68.2% of applicant PSs, while the lower one-third comprised 15.8% of ChatGPT and 9.1% of authentic PSs. ANOVA and Wilcoxon p-values (0.2757 and 0.2788, respectively) showed no statistically significant difference between ChatGPT and authentic PSs in their comparative quality across cycles.

Regarding the role of PSs on interview invitation decisions, 76.3% of respondents indicated that ChatGPT-generated PSs would have played a role, while 81.8% noted the same for authentic PSs. Conversely, 23.7% of ChatGPT PSs and 18.2% of applicant PSs were deemed unimpactful. The chi-square p-value (0.5399) reveals no statistically significant differences between the two groups concerning the impact of PSs on interview invitation decisions.

In evaluating whether the PSs alone would adversely affect respondents' decisions to invite an applicant for an interview, 13.2% of directors indicated "Yes" for ChatGPT-generated PSs, compared to 2.3% for authentic PSs. Conversely, 86.8% of ChatGPT PSs and 97.7% of authentic PSs resulted in a "No." The chi-square p-value (0.0591) suggests no statistically significant difference between the two groups in their responses.

When assessing whether interviewers felt that additional questions were needed to clarify content in the provided PSs, 26.3% of ChatGPT PSs and 22.7% of authentic PSs led to a "Yes" response, while 73.7% of ChatGPT PSs and 77.3% of authentic PSs led to a "No" response from the respondents. The chi-square p-value (0.7059) indicates no statistically significant difference between groups.

Additionally, the free-text responses were collected and collated into a document for further review (full text included in Appendix 1). Some general themes we observed from the free-text responses were that they were generic and lacked depth, overuse of flowery language, had minimal impact on decision-making, were useful for clarifying fit, and had a formulaic structure. Regardless of whether they were human- or AI-written, many responses commented on the formulaic structure, the generic nature and lack of depth, and the overuse of flowery language.

In conclusion, the statistical analyses, including ANOVA, Wilcoxon, and chi-square tests, revealed no significant differences between the two groups, suggesting that both AI-generated and authentic PSs demonstrate comparable performance in terms of grammar and writing style, personal attributes, and the utility of PSs in the interview selection process.

## Discussion

This survey, included in its entirety in Appendix 2, was distributed to the CORD Survey Community. This group, comprising over 2,500 individuals, includes program directors, associate program directors, assistant program directors, clerkship directors, and educational faculty, representing the key stakeholders in discussions regarding the application process. The "thirds scale" was used in the first three questions for both its familiarity to SLOE authors/readers, who would presumably make up the respondents, as well as its utility as a more granular assessment than a simple binary question. The survey was initiated by asking if respondents routinely performed application reviews as part of their duties and then excluded those who reported that they did not. Although the sample was heterogeneous and anonymity limited the verification of roles, the remaining group adequately represents a random sample of the diverse stakeholders in the application review process. The group surveyed received only minimal instruction, with no indication that some PSs were generated by AI, effectively blinding the respondents to the nature of the survey.

These blinded respondents evaluated the PSs with similar distributions to the thirds across both groups, strongly suggesting that they found no meaningful differences between the authentic and AI-generated (Table 1). The one category that approached a statistically significant difference was in respondent evaluation of some AI-generated PSs as adversely impacting interview invitations (p = 0.059) (Table 1). However, this result appears to be heavily influenced by the fact that almost none of the PSs were described as adversely affecting an interview invitation. The nature of the PS, lacking a defined function or goal for each reviewer and applicant, limits our ability to determine true non-inferiority among AI-generated PSs; however, the complete lack of differences in each category suggests that these experienced application reviewers found no relevant issues with the AI-generated PSs that would differentiate them from a typical applicant.

These data demonstrate that ChatGPT, with minimal editing or prompting, can generate PSs that are of comparable quality to authentic ones in the eyes of application reviewers. The implication is that significant time would be saved for the applicant, without costing them anything in terms of the perception of the PS they ultimately submit. The applications of this rapidly evolving technology are broad indeed, with the potential to shift paradigms of healthcare practice, research, and education [6,7]. However, as with any tool, there are appropriate uses and pitfalls, and this is one area where its use may be inappropriate. At their core, LLMs generate results by reusing material they have access to, making a trend towards homogeneity and repetition intrinsic to their compositions. Despite this, it would still seem naïve to think that applicants, especially those previously faced with an average of 15 hours of composition time, will not use this technology to produce these essays more efficiently. This is especially true when one considers its greater utility with more specific prompting and its constant evolution and improvement. While using LLMs for PSs may result in beneficial time savings for both applicants and even application advisors/editors, the very rapidity and trend towards homogeneity and impersonality seem to further distill the already somewhat unclear intent and utility of the PS. Program directors do not want an application season full of opaque and heterogeneous metrics and especially do not want to waste time reading generic, computer-generated essays. NRMP survey data have long suggested that the PS's utility is mixed at best, now presumably even more so since the introduction of a separate ERAS section with the stated purpose of explaining potential "red flags" in an application [5]. To that point, many of the subjective comments from the surveyed were perhaps even more descriptive than the data, demonstrating broad heterogeneity in both their opinions of the same PSs and even in their expectations for PSs in general. Some of these comments even bluntly stated the respondents’ general ambivalence or even outright distaste for PSs, with one reviewer going as far as to say, "I don't read them (PSs) to begin with, so it (PS) has no effect on my decision-making."

Given the mixed opinions on their utility and the considerable time investment required, it may be worth considering revising the PS process and exploring alternative measures that better serve the purpose. There are precedents in both business and education for other metrics of personality and fitness that align with a particular group's core values. For example, the Five Factor Model (FFM) of personality and its inventories have been widely utilized in both organized psychology and the business world for years, with longstanding support for their inter-rater reliability, even when compared to other tests, such as the Myers-Briggs Type Indicator (MBTI) [11]. The FFM has been suggested as a consideration during the recruiting season by CORD and may offer a possible consideration for some improvement/replacement of the PS model [12]. Situational judgment tests (STJs) are another area of emerging evaluation in the medical school admission process. These tests are used to assess "non-academic" qualities in an applicant and provide a metric for professionalism and other characteristics, consistently demonstrating validity and a notable lack of biases that have plagued other similar instruments [13-15]. While STJs are only just emerging in the GME application literature, there is evidence supporting the predictive ability, with minimal bias, for a prospective resident's progression through milestones, and evidence suggesting utility in predicting attrition [15,16]. Ultimately, personality inventories, STJs, and others will face some of the challenges of PSs when subjected to the rigorous gamesmanship of the residency application process. However, thoughtful consideration of how to best maximize interpersonal fits during the residency application process should be at the forefront of targets for innovation in an increasingly virtual era rife with the challenges of rapidly evolving technologies.

This study has several limitations. First, the inherently subjective nature of PS evaluation engenders a significant lack of inter-rater reliability and makes it difficult to measure quality in a fully quantitative manner. Reviewer biases, varying levels of engagement with PSs, and differences in interpretation could influence responses. Second, the survey sample was limited to members of the CORD community who voluntarily participated, potentially introducing selection bias. Additionally, the AI-generated PSs were produced using minimal prompting and were not edited or refined, which does not fully reflect how applicants might use AI tools to enhance their statements. Further studies, using the complete functionality of these programs, may yield even more significant results. However, ongoing development of this technology, use of more detailed and specific applicant prompting, and post-generation editing would almost certainly improve the statements generated. Furthermore, the small sample size and the limited number of PSs evaluated per respondent limit the conclusions that can be drawn and may also restrict generalizability. A labor-intensive task, such as a comprehensive PS review, would likely require significant respondent incentives to gather more data, which was ultimately beyond the scope of this work. Finally, while this study demonstrates that AI-generated PSs are not perceived as significantly different in quality from authentic PSs, this question does not specifically address the utility of PSs in general or the impact on individual application reviewers. Only indirect inferences, such as how this may reflect a trend towards homogeneity of PS content, can be made. As such, it does not assess their long-term implications on residency selection, applicant authenticity, or their broader role in the application process.

## Conclusions

Our findings suggest that, in the eyes of EM residency application reviewers, AI-generated PSs are not significantly different in quality from authentic PSs in terms of their grammar, writing style, and perceived conveyance of personal attributes. The lack of significant differences in how evaluators rated AI and authentic PSs raises questions about the utility of PSs in residency applications, particularly as AI tools become more sophisticated and widely accessible. The qualitative responses from application reviewers suggest a heterogeneity of opinions in both the purpose and utility of PSs in the application process. Given these findings, as well as the minimal weight program directors already place on PSs in interview decisions when compared to the time investment for students and their advisors, residency selection committees may need to reconsider their role in application review. Future studies should explore the utility of PSs in a broader context as well as alternative assessment methods that may offer a more reliable and meaningful evaluation of an applicant's fit for a program.

## Appendices

Appendix 1: aggregated free-text responses

AI-Generated Comments (#1-#5)

#1

I don't read them to begin with so it has no effect on my decision-making.

Again, extremely generic "anyone" statement - gives me no more insight into how they will function as a resident, but doesn't make me worried about training them either. The only way this would make me less likely to interview them is if there was an obvious "negative" on their application (e.g. USMLE failure, course failure) or if there was an unexplained gap in training that they otherwise should have made some effort to explain or demonstrate commitment to improvement in. 

#2

The red flag here is that it is too long. If it is over a page, I stop reading and worry the applicant should go into IM.

This personal statement has the flowery, unnecessary language. It really doesn't say much of anything specific.

Historical background discussing experience as a Paramedic helpful 

I always have questions about an applicant’s PS. 

This is an "anyone" personal statement - generic, non-offensive, attempt to insert a little quirkiness - but wouldn't sway me one way or another. Really the only personal statements for me that make a difference are for applicants who had something "bad" during their training - like a course or USMLE failure - or who had gap years in their training and expound upon what they did during those years. Or if their personal statement indicates that they may have one or more bodies hidden on their property.

#3

I don't read them to begin with so it has no effect on my decision making.

The writing style is a little flowery, but overall this is better written than most personal statements I’ve read.

Meh. It sounds cheesy. I'm in the military and work with folks who do dive medicine and no one uses phrases like "navigating the vast oceans" or "tranquil shores to the tumultuous depths of the ocean". It sounds like ChatGPT wrote this. If this student really wants to do dive medicine, he or she should be applying to the Navy EM programs and I don't see any mention of military medicine in this essay so it would make me think they just want to be out on the ocean fishing.

This one takes the deep sea analogy too far. 

For combined programs the personal statement is key to helping determine their interest and why a combined residency is important to them, as well as bring out any unique life experiences.

#4 

Very general statement, without too much specific to emergency medicine, but would not hurt the applicant. 

This piece feels manufactured a lá AI. It does have the "feel" of some personal statements I have read these last few years which relay a lot of platitudes but don't convey much of anything which I wish to know. 

The personal statement is somewhat generic with unnecessarily flowery language. 

It has too much intensity, the overuse of descriptive heavy-handed adjectives makes it difficult to read. Nearly every noun has an an adjective, and they're all extreme: fervent, unflinching, unyielding - I'm all for motivated candidates, but this sounds exhausting.

#5 

Better weittent than most and conveys more about this applicant personally, but I don’t care as much about the tranquil landscapes of their childhood as they seem to do. 

It's not going to sink them but not very relevant to EM 

I rarely am influenced by personal statements one way or the other. I like that in this example, she states she has an interest in rural health as there is such a need for rural EM physicians and it is a topic I am interested in. I also like that she's an athlete as I think there is a lot of leadership skills, teamwork, and time management needed for student athletes. That being said, personal statements are not considered that important at my institution and only really stand out if they are exceptional with a compelling story (or exceptionally bad or poorly written). 

It's effective in their commitment to rural medicine. The language suffers from overuse of heavy-handed descriptive adjectives but it's not as intense as the first example. I appreciate that it gives me insight into their career plans and I have a better understanding of what they want to do with emergency medicine. As an urban program that doesn't have any significant rural experiences, I would read this and wonder why they would want to come to me, and would likely decrease my willingness to interview. 

*Human-Generated Comments (#6-#10)* 

#6 

The personal statement came across as not particularly unique, but still well written and conveys this applicant's passion for emergency medicine. The statement would 0t hurt this applicant, but would not likely give them extra points/credit towards an interview invite. 

I would have liked the essay to begin with paragraph 3. Again I do not need the first 2 paragraphs explaining my own specialty to me. Paragraph 3 begins to describe the applicants background that influenced their choices today. I would like 2 more paragraphs expanding on this topic. The applicant is unique but I don't know enough and want to read more. 

l think the question is does this add anything to the rest of the application. In this case you have taken the personal statement out of that context which changes everything. With the CV and other information they become a lot less useful.

Aside from the specifics on being a first-generation college student and juggling multiple jobs, not much substance in here. 

This student has shown grit, perseverence, and ability. Based on personal statement alone, I suspect that they would be a star in any program. 

#7 

Would probably explore whether the applicant's interest/comfort with non-life-threatening cases in EM and be sure they're not expecting only high-adrenaline moments. 

in this essay, I feel I have an understanding of the applicant. Although I do not need the interview to clarify points, I want to talk to the applicant to expand on issues brought up in the essay. 

I wouldn't NEED to dedicate questions, but I would probably want to and it would help me hold a lively conversation with this person. 

Demonstrates an impressive degree of maturity and insight. 

This is too long. Moreover, they bounce from topic to topic. It starts out ok and then ventures too far afield. Quit trying too hard! 

This personal statement is similar to the others except that it demonstrates self-reflection and commitment to identifying and improving one's own biases, so it would actually probably make me MORE likely to interview this person if there were some other reason (e.g., scores, medical school performance) that we were on the fence. 

#8 

I don't read them to begin with, so it has no effect on my decision making. 

All over the place. Too tangential - makes me worry they are not organized. Might still interview them, butwould definitely hesitate and maybe not if a lot of other good apps. 

I always have questions about an applicant’s PS.

The way this shapes up, the tendon injury was a defining event in this student's life. While it could just be an example that was used, it does seem to focus down (even with the EMIG comments) on a limited life experience. As a result, it neither shows the depth of experience nor a grit in the face of adversity that some others might. 

It's pretty much the standard EM personal statement. Start with a personal story of trauma, make it read like the script for a movie trailer, transition awkwardly into why you chose emergency medicine. Talk about how you liked everything else but didn't love one thing, give a couple qualifiers about EM specific things you did. Its formulaic nature makes it very middle 1/3. My gut reaction is the level of drama applied to a patellar tendon rupture in a pick-up basketball game is a bit much, but the thing that stands out to me the most is the grammatical error in the EMIG statement, but if the rest of the application was strong, this wouldn't have a huge impact. 

#9 

I did not like the writing style as much as the last one, however the narrative came across as slightly more humble. This statement would neither help 0r hurt the applicant compared to all others. 

I look for paragraphs 2 and 4. I have read paragraphs 1 and 3 too often in applications. I don't want applicants to talk about the excitement of the ED, the love of procedures. I want to read about them. How do past experiences equip them to handle life in the ED. How have successes and failures influenced them. I get turned off when applicants tell me about a field I have worked in for 25 years. 

You all did a great job with this PS. it reads like 75% of the statements that I see. It is well written in a generic way that most are these days. it doesn't harm or hurt the student. 

This is your pretty standard personal statement. It is really long and I may not read the whole thing if this were not a research study. I'm not blown away by anything here but also not annoyed by anything here. I am generally not a fan of cheerleaders (my single mom always taught me to be the one being cheered for), but I thought she demonstrated empathy and clearly explained why this was her specialty choice. 

Way too long. All good words, but could condense to 2 (maybe 3) paragraphs and communicate samemessage. Would make me wonder if they are able to be organized and efficient. 

#10 

Explanation of why EM is maybe a bit generic (could almost copy/paste other specialty names for EM). Given the recent Match, might probe a bit more to try to ascertain level of commitment to EM specifically. 

Clipped writing style and boring content, but no red flags. 

I have a military bias, so would probably make me more likely to invite them.

This is a much better written personal statement than the first. Both are written by veterans but this one is far more engaging and lacks (in a good way) the flowery language of the first example.

I wouldn’t need to ask clarifying questions during the interview, but I often use the personal statement as a jumping off point for questions. I am also curious if this was generated with chatGPT - seems particularly bland language.

This Personal Statement really goes overboard trying to valiantly justify their circuitous route. They try too hard. 

Her background as an engineer is interesting, and I would want to know why she transitioned back to medicine (which had been her goal before). Being nitpicky, the sentence structure at times could have benefited from some commas. 

Appendix 2: complete survey instrument with personal statements

Start of Block: Intro Block

Q1 Do you regularly review ERAS applications for the purpose of determining interview invitations?

o Yes (1)

o No (2)

Skip To: End of Survey If Do you regularly review ERAS applications for the purpose of determining interview invitations? = No

Page Break

End of Block: Intro Block

Start of Block: B1

Q2.1 Please assess the following personal statement as if it were in an ERAS application to your program and lend your insight to the subsequent questions.

Q3.1

As a medical student hailing from the vibrant city of [redacted], my journey in medicine has been a culmination of diverse experiences, personal passions, and unwavering dedication. As I stand at the precipice of my medical career, I am thrilled to apply for your emergency medicine residency program, bringing with me a unique blend of skills, insights, and a fervent commitment to the field. Growing up amidst the bustling urban landscape of [redacted], I was exposed to the intricate tapestry of human life, diversity, and the urgency that defines the realm of emergency medicine. This exposure ignited a deep-seated fascination for the specialty - the idea of being at the forefront of medical care, making crucial decisions in critical moments, and alleviating pain and uncertainty resonated profoundly with me. As a medical student, my goal has been to transform this fascination into a lasting impact on patients and their families.

My journey has been enriched by my parallel passion for photography. Capturing moments through a lens has taught me to observe, appreciate, and empathize with the diverse stories that shape the human experience. This skill has been surprisingly congruent with my role as an emergency department scribe, where keen observation, rapid assessment, and effective communication are paramount. Through my camera's lens and in the emergency room, I have learned to preserve fleeting moments - in one, capturing the essence of a momentary scene, and in the other, capturing the essence of a patient's life-changing event. My time as an emergency department scribe was transformative. It exposed me to the dynamic and often unpredictable nature of emergency medicine. In those fast-paced environments, I witnessed the collaboration, the swift decision-making, and the unique connection between healthcare providers and patients. These experiences not only solidified my aspiration to become an emergency physician but also nurtured my ability to stay focused under pressure, communicate succinctly, and adapt to ever-changing situations. What excites me about emergency medicine is its fusion of medical acumen and human connection. Each patient arrives with a unique story, seeking help in a moment of vulnerability. I am eager to contribute by not only delivering medical expertise but also by providing comfort, assurance, and understanding. The Emergency Medicine Residency Program represents to me an opportunity to further hone my skills, broaden my perspective, and refine my approach to patient care. The comprehensive training offered by your esteemed program aligns seamlessly with my aspirations. I am drawn to the emphasis on teamwork and collaboration - values I have embraced through my photography and my experiences in the emergency room. I am committed to fostering an environment of mutual respect and effective communication to deliver the highest standard of care. In conclusion, my journey from the bustling streets of [redacted] to the fast-paced realm of emergency medicine has been an exploration of compassion, observation, and resilience. My photography has taught me to tell stories, while my time as a scribe has shown me the stories hidden within the chaos of the emergency department. I am excited to take this journey further as an emergency medicine resident, to capture moments of impact and contribute to the diverse narratives that define this field. With gratitude and enthusiasm, I look forward to the opportunity to join your program and make a meaningful difference in the lives of patients.

Q4.1 Please answer the following questions about the preceding personal statement.

How would you evaluate the quality of grammar/writing style? (1)

Lower 1/3 (1)

Middle 1/3 (2)

Upper 1/3 (3)

How well does the personal statement convey their personal attributes as a unique applicant? (2)

Lower 1/3 (1)

Middle 1/3 (2)

Upper 1/3 (3)

How does this personal statement compare to the others you have reviewed across application cycles? (3)

Lower 1/3 (1)

Middle 1/3 (2)

Upper 1/3 (3)

Q5.1 In current practice, do you utilize personal statements when making decisions to invite applicants to an interview at your program?

o Yes (1)

o No (2)

Q6.1 All other factors being equal (scores, SLOEs, MSPE, etc.), would this personal statement alone adversely affect your decision to invite this applicant for an interview?

o Yes (1)

o No (2)

Q7.1 Do you feel that you would need to dedicate interview questions to clarify any of the content of this personal statement in the subsequent interview?

o Yes (1)

o No (2)

Q8.1 (Optional) Please include any additional comments/explanations regarding this personal statement or subsequent questions.

________________________________________________________________

________________________________________________________________

________________________________________________________________

________________________________________________________________

________________________________________________________________

End of Block: B1

Start of Block: B2

Q2.2 Please assess the following personal statement as if it were in an ERAS application to your program and lend your insight to the subsequent questions.

Q3.2 In the world of medicine, I have found my true calling, a journey that seamlessly intertwines my passions, experiences, and aspirations. As a dedicated medical student hailing from [redacted], I am excited to apply for your emergency medicine residency program, where I hope to channel my diverse background, personal interests, and unwavering commitment into a thriving career. [redacted] vibrant community and ever-evolving healthcare landscape have kindled my drive to become an emergency medicine physician. The city's resilience mirrors my own determination to provide immediate care and support during critical moments in patients' lives. This journey, however, goes beyond the confines of a bustling urban environment; it extends to the serene waters where I find solace and inspiration - in fly fishing. Fly fishing, with its delicate balance of technique and patience, mirrors the essence of emergency medicine. Just as the fly dancer lures the fish, the emergency physician must entwine medical knowledge with a calming presence to navigate the complexities of patient care. In the serene rivers and amidst the chaos of the emergency room, I have learned to be adaptive, intuitive, and composed under pressure. My prior role as a paramedic unveiled the profound impact that emergency medical care has on individuals and communities. Those moments on the front lines solidified my commitment to fostering resilience, both within myself and the patients I served. As a paramedic, I became adept at thinking on my feet, making rapid decisions, and effectively communicating in high-stress situations - skills that I carry with me into the medical field. My experiences have shaped my approach to medicine. I aspire to be a bridge between the swift world of emergency care and the compassionate realm of healing. Just as fly fishing requires a deep understanding of the water's currents and the fish's behavior, I aim to understand the nuances of each patient's needs and tailor my care accordingly. Whether through a deftly cast line or a carefully chosen treatment plan, I believe in the power of precision and attention to detail. The emergency medicine residency program at your institution aligns perfectly with my values and aspirations. Its reputation for fostering a collaborative and diverse learning environment resonates with my background as a paramedic and my passion for connecting with individuals from various walks of life. The emphasis on teamwork, rapid decision-making, and comprehensive training aligns seamlessly with my vision of becoming an effective and compassionate emergency medicine physician. In conclusion, my journey from [redacted] bustling streets to the tranquil rivers and the high adrenaline setting of a paramedic has shaped my resolve to contribute to the dynamic field of emergency medicine. Through fly fishing, I have learned the harmony of patience and precision, mirroring the essence of this discipline. I am eager to translate these skills, experiences, and values into an impactful residency experience, to offer unwavering support and immediate care to those who need it most. With genuine enthusiasm and a readiness to embrace challenges, I am excited about the possibility of joining your program and contributing to its legacy of excellence.

Q4.2 Please answer the following questions about the preceding personal statement.

How would you evaluate the quality of grammar/writing style? (1)

Lower 1/3 (1)

Middle 1/3 (2)

Upper 1/3 (3)

How well does the personal statement convey their personal attributes as a unique applicant? (2)

Lower 1/3 (1)

Middle 1/3 (2)

Upper 1/3 (3)

How does this personal statement compare to the others you have reviewed across application cycles? (3)

Lower 1/3 (1)

Middle 1/3 (2)

Upper 1/3 (3)

Q5.2 In current practice, do you utilize personal statements when making decisions to invite applicants to an interview at your program?

o Yes (1)

o No (2)

Q6.2 All other factors being equal (scores, SLOEs, MSPE, etc.), would this personal statement alone adversely affect your decision to invite this applicant for an interview?

o Yes (1)

o No (2)

Q7.2 Do you feel that you would need to dedicate interview questions to clarify any of the content of this personal statement in the subsequent interview?

o Yes (1)

o No (2)

Q8.2 (Optional) Please include any additional comments/explanations regarding this personal statement or subsequent questions.

________________________________________________________________

________________________________________________________________

________________________________________________________________

________________________________________________________________

________________________________________________________________

End of Block: B2

Start of Block: B3

Q2.3 Please assess the following personal statement as if it were in an ERAS application to your program and lend your insight to the subsequent questions.

Q3.3 In my journey through medicine, I've discovered that the art of healing is much like navigating the vast oceans-both demand unwavering dedication, adaptability, and a deep respect for the unknown. As a medical student hailing from [redacted], I am thrilled to embark on the next phase of my journey by applying for your emergency medicine residency program. My diverse background, coupled with a passion for dive medicine and a unique experience as a deep-sea fishing boat captain, has instilled in me a profound appreciation for resilience, quick thinking, and providing immediate care. Growing up in the coastal charm of [redacted], I've felt a deep connection to both the serene beauty and unpredictability of the ocean. My years as a deep-sea fishing boat captain were a masterclass in adaptability-navigating ever-changing waters, responding to unpredictable weather, and leading my crew with a steady hand. These experiences have uniquely prepared me for the demanding nature of emergency medicine, where rapid decisions and the ability to stay calm under pressure are paramount. My fascination with dive medicine has been a driving force in my medical journey. The parallel between exploring the ocean's depths and delving into the complexities of medicine is unmistakable. Both fields require meticulous preparation, acute awareness, and a willingness to dive into the unknown. Just as I ensured the safety and well-being of divers, I am dedicated to providing the same level of care to patients in their moments of need. As a former deep-sea fishing boat captain, I have been entrusted with the lives of those under my care-a responsibility I approach with utmost seriousness. This experience has honed my communication skills, fine-tuned my ability to manage crises, and instilled a profound empathy for individuals facing challenging situations. These attributes, forged in the midst of the sea's unpredictable temperament, align seamlessly with the qualities required of an exceptional emergency medicine physician. The emergency medicine residency program at your esteemed institution resonates deeply with my values and aspirations. Its emphasis on rapid decision-making, interdisciplinary collaboration, and comprehensive training reflects my own desire to provide effective and compassionate care. The dynamic nature of emergency medicine aligns perfectly with my background, and I am eager to embrace the challenges it presents. In conclusion, my journey from [redacted] tranquil shores to the tumultuous depths of the ocean has shaped my unwavering commitment to providing immediate, compassionate care to those in need. My passion for dive medicine, coupled with the skills honed as a deep-sea fishing boat captain, has instilled in me a unique perspective on patient care. With enthusiasm and dedication, I look forward to the opportunity of joining your program, where I can combine my experiences, my passions, and my unwavering commitment to becoming a proficient emergency medicine physician.

Q4.3 Please answer the following questions about the preceding personal statement.

How would you evaluate the quality of grammar/writing style? (1)

Lower 1/3 (1)

Middle 1/3 (2)

Upper 1/3 (3)

How well does the personal statement convey their personal attributes as a unique applicant? (2)

Lower 1/3 (1)

Middle 1/3 (2)

Upper 1/3 (3)

How does this personal statement compare to the others you have reviewed across application cycles? (3)

Lower 1/3 (1)

Middle 1/3 (2)

Upper 1/3 (3)

Q5.3 In current practice, do you utilize personal statements when making decisions to invite applicants to an interview at your program?

o Yes (1)

o No (2)

Q6.3 All other factors being equal (scores, SLOEs, MSPE, etc.), would this personal statement alone adversely affect your decision to invite this applicant for an interview?

o Yes (1)

o No (2)

Q7.3 Do you feel that you would need to dedicate interview questions to clarify any of the content of this personal statement in the subsequent interview?

o Yes (1)

o No (2)

Q8.3 (Optional) Please include any additional comments/explanations regarding this personal statement or subsequent questions.

________________________________________________________________

________________________________________________________________

________________________________________________________________

________________________________________________________________

________________________________________________________________

End of Block: B3

Start of Block: B4

Q2.4 Please assess the following personal statement as if it were in an ERAS application to your program and lend your insight to the subsequent questions.

Q3.4 In the dynamic landscape of medicine, I have discovered my true calling-an intersection of my passions, experiences, and an unwavering commitment to serving others. Hailing from the captivating town of [redacted], I am excited to submit my application for your emergency medicine residency program. My unique blend of experiences, coupled with a fervent interest in mountain biking and a foundation as a military veteran, has fortified my resilience, sharpened my decision-making skills, and steeled my dedication to thriving under pressure. [Redacted] majestic mountains have been both a sanctuary and a classroom-a place where I've nurtured a deep-seated passion for mountain biking. The harmony of challenge and serenity inherent in mountain biking mirrors the ethos of emergency medicine. Just as I navigate treacherous trails, I am drawn to navigating the intricate path of emergency care, making split-second decisions, and ensuring a safe passage for those in need. My journey took an unconventional turn when I served in the military. This role not only bestowed upon me an unwavering commitment to service and discipline but also taught me the gravity of quick decision-making in life-or-death situations. These experiences in the military refined my ability to thrive under duress, to communicate effectively, and to remain composed amid chaos-traits that have become a cornerstone of my approach to medicine. My interest in emergency medicine is a natural extension of my military service-an opportunity to continue serving and making an immediate impact. The structured environment of the military and the unpredictable nature of emergency medicine both demand resilience, adaptability, and a relentless commitment to the well-being of others. Whether it's a demanding mountain trail or a high-pressure emergency room, I am unflinchingly dedicated to delivering my best. The emergency medicine residency program at your institution embodies the values I hold dear. Its emphasis on rapid decision-making, teamwork, and comprehensive training align seamlessly with my military background and my fervor for mountain biking. The diverse patient population and commitment to providing top-notch care in critical moments deeply resonate with my aspirations. In closing, my journey from the tranquil mountains of [redacted] to the rigors of military service has forged in me an unyielding dedication to excellence, resilience, and quick decision-making. My passion for mountain biking reflects my love for challenge, growth, and pushing boundaries-a mindset I bring into the field of medicine. With eagerness and commitment, I am excited about the possibility of joining your program and contributing to its legacy of excellence in emergency medicine.

Q4.4 Please answer the following questions about the preceding personal statement.

How would you evaluate the quality of grammar/writing style? (1)

Lower 1/3 (1)

Middle 1/3 (2)

Upper 1/3 (3)

How well does the personal statement convey their personal attributes as a unique applicant? (2)

Lower 1/3 (1)

Middle 1/3 (2)

Upper 1/3 (3)

How does this personal statement compare to the others you have reviewed across application cycles? (3)

Lower 1/3 (1)

Middle 1/3 (2)

Upper 1/3 (3)

Q5.4 In current practice, do you utilize personal statements when making decisions to invite applicants to an interview at your program?

o Yes (1)

o No (2)

Q6.4 All other factors being equal (scores, SLOEs, MSPE, etc.), would this personal statement alone adversely affect your decision to invite this applicant for an interview?

o Yes (1)

o No (2)

Q7.4 Do you feel that you would need to dedicate interview questions to clarify any of the content of this personal statement in the subsequent interview?

o Yes (1)

o No (2)

Q8.4 (Optional) Please include any additional comments/explanations regarding this personal statement or subsequent questions.

________________________________________________________________

________________________________________________________________

________________________________________________________________

________________________________________________________________

________________________________________________________________

End of Block: B4

Start of Block: B5

Q2.5 Please assess the following personal statement as if it were in an ERAS application to your program and lend your insight to the subsequent questions.

Q3.5 In the realm of medicine, I have discovered my true purpose-a harmonious fusion of my passions, experiences, and a resolute commitment to making a meaningful impact. Hailing from the charming town of [redacted], I am eager to submit my application for your emergency medicine residency program. With a unique blend of experiences, a deep-rooted interest in rural healthcare, and a foundation as a former collegiate soccer player, I am well-equipped to excel in the fast-paced and high-pressure field of emergency medicine. Growing up in the vast landscapes of [redacted], I developed a profound appreciation for the power of close-knit communities and their unique healthcare needs. This background, coupled with my unwavering commitment to rural healthcare, has fueled my drive to bring top-tier medical care to underserved areas. Just as I've witnessed the unity and resilience of [redacted] communities, I am dedicated to being a steadfast source of support for patients during their most critical moments. My experience as a former collegiate soccer player has equipped me with invaluable skills that seamlessly translate to emergency medicine. The ability to think on my feet, communicate effectively under pressure, and work harmoniously in a team environment are qualities that have been honed on the field and are crucial in delivering optimal patient care. Soccer taught me that each team member plays a vital role in the collective success-a philosophy I carry with me into medicine. My passion for rural healthcare is deeply intertwined with my commitment to serving communities that often lack access to specialized medical resources. I am drawn to the challenges and rewards that come with delivering care in remote areas, where immediate medical attention can be a lifeline. The idea of becoming a physician who not only provides treatment but also empowers individuals to take control of their health resonates deeply with me. The emergency medicine residency program at your esteemed institution perfectly aligns with my aspirations and values. Its emphasis on rapid decision-making, interdisciplinary collaboration, and comprehensive training mirrors my dedication to providing exceptional care in rural settings. The program's commitment to diversity and its mission to serve underserved populations resonate deeply with my own goals. In conclusion, my journey from the tranquil landscapes of [redacted] to the rigor of collegiate soccer has equipped me with a unique set of skills and values that align seamlessly with the demands of emergency medicine. My passion for rural healthcare drives my commitment to making a lasting impact on communities that often face healthcare disparities. With unwavering enthusiasm, I am excited about the possibility of joining your program and contributing to its legacy of excellence in emergency medicine.

Q4.5 Please answer the following questions about the preceding personal statement.

How would you evaluate the quality of grammar/writing style? (1)

Lower 1/3 (1)

Middle 1/3 (2)

Upper 1/3 (3)

How well does the personal statement convey their personal attributes as a unique applicant? (2)

Lower 1/3 (1)

Middle 1/3 (2)

Upper 1/3 (3)

How does this personal statement compare to the others you have reviewed across application cycles? (3)

Lower 1/3 (1)

Middle 1/3 (2)

Upper 1/3 (3)

Q5.5 In current practice, do you utilize personal statements when making decisions to invite applicants to an interview at your program?

o Yes (1)

o No (2)

Q6.5 All other factors being equal (scores, SLOEs, MSPE, etc.), would this personal statement alone adversely affect your decision to invite this applicant for an interview?

o Yes (1)

o No (2)

Q7.5 Do you feel that you would need to dedicate interview questions to clarify any of the content of this personal statement in the subsequent interview?

o Yes (1)

o No (2)

Q8.5 (Optional) Please include any additional comments/explanations regarding this personal statement or subsequent questions.

________________________________________________________________

________________________________________________________________

________________________________________________________________

________________________________________________________________

________________________________________________________________

End of Block: B5

Start of Block: B6

Q2.6 Please assess the following personal statement as if it were in an ERAS application to your program and lend your insight to the subsequent questions.

Q3.6 The call for the trauma came in, each team member silently assuming their assigned position. It was clear that an unspoken code had been activated amongst the Emergency Department staff. The choreographed dance began as the patient was brought in, each individual role integrating perfectly with the others into a synchrony unlike anything I had ever seen before. In a time when others might feel overwhelmed by the commotion, I felt at peace watching the team work together seamlessly to stabilize the patient. Suddenly I knew for certain this was something I wanted to be a part of. It did not take long to learn that I have never felt as alive as I do in the Emergency Department. Despite thriving on routine at home, walking into the hospital not knowing what is going to come my way in the next several hours has me excited to take on the day. There is a thrill with each case, major or minor, that forces me to apply and improve upon my medical skills and knowledge on the spot that I believe will never go away. Every patient is an opportunity to diversify my abilities and grow as a future physician. I will excel as an Emergency Medicine physician because I work well under pressure and am able to balance many responsibilities, modifying my course as needed when something unpredictable occurs. As a first-generation college student, I completed both my Bachelor’s and Master’s degrees during the time most others complete their undergraduate degree alone. I worked three jobs while studying for and passing those classes. The work ethic I developed through those experiences has carried me through medical school and will continue to carry me into my career as a physician. The leadership skills I gained while being a campus tour guide and holding executive positions in student organizations during medical school will allow me to better promote patient care by leading the care team towards the ultimate goal of serving the patient safely and effectively. While there is something to be said for continuity of care, I believe the nature of Emergency Medicine provides its own unique opportunities to create a meaningful rapport with patients. The physician must read the patient and establish a trusting relationship within a matter of moments in order to best assess the patient and their needs. To me, that initial interaction poses a distinct yet rewarding challenge of its own, something separate from healthcare but arguably just as, if not more, important. I enjoy getting to know several patients in a short span of time and helping them feel more at ease during some of their most vulnerable moments. I entered medical school wanting to be an Emergency Medicine physician, and through clinical rotations my passion for the field has only grown. I want my career to embody as much of the vastness of medicine as possible, allowing me to perform procedures and investigate different pathologies all while having the privilege of being the first person to piece together the puzzle of what might be causing a patient’s distress in their time of need. I believe my curiosity about medicine, compassion, and ability to adapt to the unexpected will make me a valuable member of this residency program and successful future Emergency Medicine physician, and I am excited to see where this opportunity takes me.

Q4.6 Please answer the following questions about the preceding personal statement.

How would you evaluate the quality of grammar/writing style? (1)

Lower 1/3 (1)

Middle 1/3 (2)

Upper 1/3 (3)

How well does the personal statement convey their personal attributes as a unique applicant? (2)

Lower 1/3 (1)

Middle 1/3 (2)

Upper 1/3 (3)

How does this personal statement compare to the others you have reviewed across application cycles? (3)

Lower 1/3 (1)

Middle 1/3 (2)

Upper 1/3 (3)

Q5.6 In current practice, do you utilize personal statements when making decisions to invite applicants to an interview at your program?

o Yes (1)

o No (2)

Q6.6 All other factors being equal (scores, SLOEs, MSPE, etc.), would this personal statement alone adversely affect your decision to invite this applicant for an interview?

o Yes (1)

o No (2)

Q7.6 Do you feel that you would need to dedicate interview questions to clarify any of the content of this personal statement in the subsequent interview?

o Yes (1)

o No (2)

Q8.6 (Optional) Please include any additional comments/explanations regarding this personal statement or subsequent questions.

________________________________________________________________

________________________________________________________________

________________________________________________________________

________________________________________________________________

________________________________________________________________

End of Block: B6

Start of Block: B7

Q2.7 Please assess the following personal statement as if it were in an ERAS application to your program and lend your insight to the subsequent questions.

Q3.7 Growing up in a rural area, I learned firsthand that emergency medicine can become the primary-and sometimes only-way many people interact with the healthcare system. In college, I started to explore the specialty further through volunteer work spending many hours wheeling patients to the “donut of truth” and participating in the customer service aspect of healthcare. In 2018, I became fully immersed in the world of emergency medicine, working as a scribe alongside physicians in a level 1 trauma center. Approximately 2,700 patients later, I left for medical school with the goal to someday return to the emergency department as a resident physician. Throughout medical school, I sharpened my research skills and developed a passion for mentorship, becoming involved with my school’s advising programs and interview team. My clerkship year spent in [redacted] was invaluable, giving me the opportunity to experience an urban hospital environment with medical and socioeconomic challenges very different from the [redacted] region in which I grew up. Despite the different circumstances of my new surroundings, coming back to the emergency department as a rotating medical student felt like home. In my opinion, the best part about becoming an emergency medicine physician is achieving the training and skills required to save a patient’s life despite frequent constraints on available history. During my rotation in a community hospital, a woman was brought by ambulance in cardiac arrest who reportedly had left sided weakness prior to the event - there was no other history available to us. At first, we were concerned for a possible brain herniation, but during resuscitation we were informed that she was a dialysis patient who had missed her recent appointments. We quickly administered medications to correct for possible hyperkalemia and were able to successfully stabilize the patient. Not only was this my first time successfully intubating and placing a central line in the emergency department, but I learned from this experience the importance of avoiding anchoring bias while keeping a broad differential with every patient you encounter. My experiences prior to and during medical school have given me a front row seat to the most rewarding but also the most challenging days of a resident’s career. I remember the lives saved, but even more so I remember the lives lost and how the residents responded to these tragedies. Just as important, I came to understand the impact that one’s attitude moving forward has on subsequent patient care. My ability to discern between healthy and self-destructive ways of handling losses and failures will help me to avoid lasting impacts of emotional fatigue and burnout while striving to flourish in an environment of constant interruptions. One of the ways in which I keep my own body and mind healthy is by long distance trail running. The steep uphill climbs, the uncertainty of the environment, and the necessity of endurance have far more to offer than a predictable flat road. Emergency medicine reflects the unpredictable nature of the trail, and I find myself drawn to this challenging, yet rewarding specialty. One minute might have you sedating a toddler to remove chunks of glass from an overturned fish tank and the next you could be telling a patient with abdominal pain for 6 months that they are actually pregnant. In Middlemarch, George Elliot writes that medicine is “the most perfect interchange between science and art; offering the most direct alliance demanded between intellectual conquest and the social good.” I’ve found that emergency medicine embodies this description perfectly, as it coalesces the practice of treating patients at their greatest time of need with an abundance of opportunities to teach and discover. My ultimate goal is to become a leader in emergency care and clinical teaching, while always striving to advocate for my patients. I am searching for a program that encompasses a spirit of education, collaboration with other specialties, and above all else a priority to improve patient care outcomes in the emergency department.

Q4.7 Please answer the following questions about the preceding personal statement.

How would you evaluate the quality of grammar/writing style? (1)

Lower 1/3 (1)

Middle 1/3 (2)

Upper 1/3 (3)

How well does the personal statement convey their personal attributes as a unique applicant? (2)

Lower 1/3 (1)

Middle 1/3 (2)

Upper 1/3 (3)

How does this personal statement compare to the others you have reviewed across application cycles? (3)

Lower 1/3 (1)

Middle 1/3 (2)

Upper 1/3 (3)

Q5.7 In current practice, do you utilize personal statements when making decisions to invite applicants to an interview at your program?

o Yes (1)

o No (2)

Q6.7 All other factors being equal (scores, SLOEs, MSPE, etc.), would this personal statement alone adversely affect your decision to invite this applicant for an interview?

o Yes (1)

o No (2)

Q7.7 Do you feel that you would need to dedicate interview questions to clarify any of the content of this personal statement in the subsequent interview?

o Yes (1)

o No (2)

Q8.7 (Optional) Please include any additional comments/explanations regarding this personal statement or subsequent questions.

________________________________________________________________

________________________________________________________________

________________________________________________________________

________________________________________________________________

________________________________________________________________

End of Block: B7

Start of Block: B8

Q2.8 Please assess the following personal statement as if it were in an ERAS application to your program and lend your insight to the subsequent questions.

Q3.8 Perspiration beads above my brow as I hobble on crutches through the wards of the hospital for morning rounds. Nine days prior, my life had changed in an instant. Saturday morning basketball with my medical school colleagues provided excellent stress relief, however, this ordinary pick-up game in July would alter my outlook for the remainder of my clinical years in school. As I attempted a shot, my knee buckled, and excruciating pain ensued. A quick glance down and the injury was apparent as my patella was closer to my hip than my knee. I was rushed to the local emergency department where I received urgent treatment from the entire team and was diagnosed with a complete patellar tendon rupture. Without the immediate care provided by the emergency department physician, I would not have been able to have a timely surgical repair that allowed me to quickly return to clinical rotations. Throughout my clerkships during the third year of medical school, I noticed that I was not particularly drawn toward one specific subset of medicine, but rather thoroughly enjoyed each discipline and how they are interconnected. Each patient has a different story, issue, or diagnosis. I found myself naturally drawn to patients with an acute illness or injury that required more rapid treatment. During encounters in which the patient had an acute issue, I could not help but think how their normal day was “turned upside down” in a similar way to mine when I ruptured my patellar tendon, and what difficulties may be waiting for them in the future. Moving forward in my medical career, I aspire to not only encounter a wide variety of diagnoses every day, but also to provide compassionate care to those with particularly acute or traumatic issues that need emergent attention. Treating acute, varying diagnoses has been exciting throughout medical school, especially while volunteering dozens of hours for [redacted] Medical Outreach which strives to serve the underserved and homeless in the community each month. As president of the emergency medicine interest group, I have had the pleasure of participating in and leading programs for other medical students, such as suture clinics and a procedure clinic involving cadavers. Gaining these skills early in one’s medical education provided an easier transition from basic science courses to the clinical years. Being able to suture the numerous lacerations that come through the door has proved invaluable to obtain experience honing one’s skills. Rehabilitation from a traumatic injury and limping with a straight-leg brace for 12 weeks in the midst of clerkships required tremendous focus, dedication, and perseverance. I juggled weekly physical therapy, clinical work, and board studying all while improving my test scores. While working with emergency medicine physicians over the last year, they have continuously demonstrated the ability to manage multiple patients simultaneously while prioritizing the most critical issues. Being in countless situations during medical school where one must think quickly and decisively has prepared me to pursue this next step in my medical journey. Much of what I had to learn because of my patellar rupture, such as focus, juggling multiple demands, and dedication to medicine even in the face of adversity, are all qualities of a good emergency medicine physician. The last four years of medical school have enhanced my drive to succeed as a physician in the near future. From late nights studying for countless exams to learning tremendous amounts of information each day during my clerkships, I have inched closer to my dream. Through a combination of knowledge from the textbooks, hands-on skills from clinical rotations, and life experiences, such as my knee injury, that have taught me strength and perseverance, I trust that I am prepared to take the next step to become an Emergency Medicine Physician.

Q4.8 Please answer the following questions about the preceding personal statement.

How would you evaluate the quality of grammar/writing style? (1)

Lower 1/3 (1)

Middle 1/3 (2)

Upper 1/3 (3)

How well does the personal statement convey their personal attributes as a unique applicant? (2)

Lower 1/3 (1)

Middle 1/3 (2)

Upper 1/3 (3)

How does this personal statement compare to the others you have reviewed across application cycles? (3)

Lower 1/3 (1)

Middle 1/3 (2)

Upper 1/3 (3)

Q5.8 In current practice, do you utilize personal statements when making decisions to invite applicants to an interview at your program?

o Yes (1)

o No (2)

Q6.8 All other factors being equal (scores, SLOEs, MSPE, etc.), would this personal statement alone adversely affect your decision to invite this applicant for an interview?

o Yes (1)

o No (2)

Q7.8 Do you feel that you would need to dedicate interview questions to clarify any of the content of this personal statement in the subsequent interview?

o Yes (1)

o No (2)

Q8.8 (Optional) Please include any additional comments/explanations regarding this personal statement or subsequent questions.

________________________________________________________________

________________________________________________________________

________________________________________________________________

________________________________________________________________

________________________________________________________________

End of Block: B8

Start of Block: B9

Q2.9 Please assess the following personal statement as if it were in an ERAS application to your program and lend your insight to the subsequent questions.

Q3.9 My first introduction to the Emergency Department was while shadowing as a timid pre-medical student during a summer medical program. Abruptly over the intercom, “Code blue, room 64”. Within seconds, an entire team was assembled. I watched with admiration as the emergency physician began issuing orders while continuously surveying various monitors. A nurse started chest compressions, a resident assisted in airway management, and another nurse began administering medication. This being my initial exposure to a medical code, I retreated to the wall. Time seemed to halt during those fearful minutes until a stable rhythm was achieved. I was captivated by how effectively the team worked together and by the confidence and calm demeanor of the physician as they deferred to her. Through the frantic, yet flawlessly organized, chaos, I realized that this dedicated team was the glue connecting patients to the care they need. Without the team’s knowledge and skill, patients don’t survive to see surgeons, cardiologists, or other necessary future providers. I came home from the hospital that day and knew that medicine, specifically emergency medicine, was the right path for me. Soon after starting my first year of medical school, I unexpectedly found myself back in the Emergency Department. My beloved grandmother was brought in from home by EMS with respiratory failure from pneumonia. Amidst the whirlwind of emotion, I vividly remember the meticulous and caring way the doctors attended to my grandmother and to us. They were able to stabilize her and admit her to the Intensive Care Unit. Despite the efforts of her medical team, she passed away peacefully two weeks later. In time, I realized that the emergency providers gave us a precious gift. Without their diligence and quick action, my grandmother’s end-of-life would have been vastly different. They gave her the opportunity to pass comfortably, surrounded by those she loved. It was clear to me then that the impact of the emergency physician lasts far beyond the emergency department, even if that provider doesn’t always see the outcome. My resolve to become an emergency physician only grew throughout my subsequent emergency medicine clerkships. I soon found myself thriving in the unpredictable, fast-paced environment. On one of my first shifts, I cared for a cirrhotic patient who needed a paracentesis, an elderly gentleman on blood thinners who had fallen at home, and a patient presenting with a COPD exacerbation, and I assisted in a blunt trauma case from a motor vehicle crash. I was immediately enthralled with the uncertainty that comes with working in the Emergency Department, as it requires quick thinking and preparedness for any illness that might come through the door. My experiences in emergency medicine since have shown me that I enjoy the unique challenges of the undifferentiated patient. Emergency medicine gives me the opportunity to use the entirety of my medical knowledge and skill to quickly assess and diagnose patients based on chief complaints. Each patient I continue to encounter in the emergency department setting can bring anything from a complicated diagnosis or an urgent life-saving intervention to the opportunity to bond with a patient during one of their darkest hours. I have identified numerous attributes that are necessary to being a great emergency physician. Having a strong work ethic, being team oriented, and showing resilience are all paramount in this field. My own work ethic was instilled in me as a child while spending long days in the garden with my grandfather, where he bestowed upon me the jobs of watering, planting and harvesting. He taught me that the harder you work, the more enjoyable the fruits of your labor will be. Participation in athletics taught me how to be a team player and the importance of group communication. Through competitive cheer and gymnastics, I learned that a team reaches their goal most efficiently if everyone does their part and communicates effectively. And my resiliency stems from growing up with a single mother. Watching her taught me the importance of independence, resourcefulness, and adaptability. Getting comfortable with change helped prepare me for life’s unexpected curve balls. My background, work ethic, and desire to succeed will make me a confident and capable emergency medicine physician. When reflecting on my journey, I am thankful for the knowledge and confidence that my medical education and personal experiences have provided. Rather than retreating to the sideline, I now play an active role during the care of my patients. Because of my grandmother’s illness, I am better able to empathize with patients and their families during critical times to offer them the same sincerity and compassion that my own family received. Emergency medicine gives me the opportunity to work with diverse patient populations and to incorporate problem solving skills to treat the entire spectrum of acute illness and injury, regardless of patient background, age, or ability to pay. My desire to advocate for my patients and my enthusiasm for the specialty fuels my dream to become a great emergency medicine physician.

Q4.9 Please answer the following questions about the preceding personal statement.

How would you evaluate the quality of grammar/writing style? (1)

Lower 1/3 (1)

Middle 1/3 (2)

Upper 1/3 (3)

How well does the personal statement convey their personal attributes as a unique applicant? (2)

Lower 1/3 (1)

Middle 1/3 (2)

Upper 1/3 (3)

How does this personal statement compare to the others you have reviewed across application cycles? (3)

Lower 1/3 (1)

Middle 1/3 (2)

Upper 1/3 (3)

Q5.9 In current practice, do you utilize personal statements when making decisions to invite applicants to an interview at your program?

o Yes (1)

o No (2)

Q6.9 All other factors being equal (scores, SLOEs, MSPE, etc.), would this personal statement alone adversely affect your decision to invite this applicant for an interview?

o Yes (1)

o No (2)

Q7.9 Do you feel that you would need to dedicate interview questions to clarify any of the content of this personal statement in the subsequent interview?

o Yes (1)

o No (2)

Q8.9 (Optional) Please include any additional comments/explanations regarding this personal statement or subsequent questions.

________________________________________________________________

________________________________________________________________

________________________________________________________________

________________________________________________________________

________________________________________________________________

End of Block: B9

Start of Block: B10

Q2.10 Please assess the following personal statement as if it were in an ERAS application to your program and lend your insight to the subsequent questions.

Q3.10 What do emergency physicians and engineers have in common? Both enjoy using a combination of cognitive and hands-on skills to solve problems. For much of my life I have found great satisfaction in overcoming challenges. Medical problems particularly interest me, and I have wanted to become a physician since the seventh grade. Even though I intended to pursue medicine, I completed a degree in engineering. It may seem an unusual choice, but I admired the way engineers design, understand, and troubleshoot complex systems and felt confident that I could apply the same approaches to medicine. After graduating from college I spent the next two years working as an electrical engineer designing instrumentation and control systems for the U.S. Navy’s nuclear submarines. Although not directly related to medicine, I consider this time to be one of the most valuable experiences in my life. As an engineer I frequently was assigned complex projects with which I had no prior experience. These assignments taught me to break problems down to simpler components, apply principles I had studied, and then revise my approach as new data became available. When I entered medical school, I kept an open mind and explored a number of specialties. Many of them interested me, but the excitement of emergency medicine always had a certain appeal. Early on during my third year I had the opportunity to take a two-week emergency medicine elective. I was amazed to find that emergency medicine shared many aspects with engineering. Patients often had complex medical histories and multiple problems requiring me to break the clinical presentation into simpler components much the way I would with a new engineering project. With guidance from my attending physicians, I created differential diagnoses, ordered tests, and implemented treatment plans. I especially enjoyed the occasions where we would develop a treatment plan and then use our hands-on skills to carry it out - whether that was suturing a laceration, incising and draining an abscess, or placing a central line. My fourth year emergency medicine rotations have solidified my interest in the field. The shifts fly by as I see interesting patients and learn to recognize new patient presentations. I have had the opportunity to take more responsibility for patient care. Following up with patients and test results and performing procedures independently makes me feel engaged and valuable to the patient and the team. The satisfaction and enjoyment I have experienced have reassured me that emergency medicine is the right field for me. I am focused on entering a residency program that will prepare me with the skills and experience needed to excel as an emergency physician. I have not yet decided what to pursue after residency. I have much to experience these next few years and am interested to learn more about various fellowship pathways and career opportunities. No matter what I decide to do next, I look forward to the exciting years ahead of building my knowledge and skills so I can solve challenging problems for patients and improve or maybe even save their lives.

Q4.10 Please answer the following questions about the preceding personal statement.

How would you evaluate the quality of grammar/writing style? (1)

Lower 1/3 (1)

Middle 1/3 (2)

Upper 1/3 (3)

How well does the personal statement convey their personal attributes as a unique applicant? (2)

Lower 1/3 (1)

Middle 1/3 (2)

Upper 1/3 (3)

How does this personal statement compare to the others you have reviewed across application cycles? (3)

Lower 1/3 (1)

Middle 1/3 (2)

Upper 1/3 (3)

Q5.10 In current practice, do you utilize personal statements when making decisions to invite applicants to an interview at your program?

o Yes (1)

o No (2)

Q6.10 All other factors being equal (scores, SLOEs, MSPE, etc.), would this personal statement alone adversely affect your decision to invite this applicant for an interview?

o Yes (1)

o No (2)

Q7.10 Do you feel that you would need to dedicate interview questions to clarify any of the content of this personal statement in the subsequent interview?

o Yes (1)

o No (2)

Q8.10 (Optional) Please include any additional comments/explanations regarding this personal statement or subsequent questions.

________________________________________________________________

________________________________________________________________

________________________________________________________________

________________________________________________________________

________________________________________________________________

End of Block: B1

## References

[1] (2025). Students & residents. Personal statement. https://students-residents.aamc.org/applying-residencies-eras/publication-chapters/personal-statement.

[2] Granger CJ, Cohen LL, Boden AL, Hasan LK, Mulcahey MK, Aiyer AA (2022). Analyzing the orthopedic surgery personal statement: do residency applicants see value in its use?. J Surg Orthop Adv.

[3] Ponterio JM, Levy L, Lakhi NA (2022). Evaluation of the virtual interviews for resident recruitment due to COVID-19 travel restrictions: a nationwide survey of US senior medical students. Fam Med.

[4] White BA, Sadoski M, Thomas S, Shabahang M (2012). Is the evaluation of the personal statement a reliable component of the general surgery residency application?. J Surg Educ.

[5] (2025). National Resident Matching Program. Results of the 2021 NRMP Program Director Survey. https://www.nrmp.org/wp-content/uploads/2021/11/2021-PD-Survey-Report-for-WWW.pdf..

[6] Hasanein AM, Sobaih AE (2023). Drivers and consequences of ChatGPT use in higher education: key stakeholder perspectives. Eur J Investig Health Psychol Educ.

[7] Sallam M (2023). ChatGPT utility in healthcare education, research, and practice: systematic review on the promising perspectives and valid concerns. Healthcare (Basel).

[8] Johnstone RE, Neely G, Sizemore DC (2023). Artificial intelligence software can generate residency application personal statements that program directors find acceptable and difficult to distinguish from applicant compositions. J Clin Anesth.

[9] Zumsteg JM, Junn C (2023). Will ChatGPT match to your program?. Am J Phys Med Rehabil.

[10] (2025). SAS/STAT 9.4 user's guide. Cary, NC: SAS Institute Inc.

[11] McCrae RR, Costa PT Jr (1989). Reinterpreting the Myers-Briggs Type Indicator from the perspective of the five-factor model of personality. J Pers.

[12] (2025). Council of Residency Directors. CORD Connects: how to recruit the best residents: lessons from the business world. https://www.youtube.com/watch.

[13] Patterson F, Zibarras L, Ashworth V (2016). Situational judgement tests in medical education and training: research, theory and practice: AMEE guide no. 100. Med Teach.

[14] Webster ES, Paton LW, Crampton PE, Tiffin PA (2020). Situational judgement test validity for selection: a systematic review and meta-analysis. Med Educ.

[15] Smith KJ, Reed BN, Neely S, Farland MZ, Haines ST, Robinson JD (2023). Opening the black box: agreement and reliability of a situational judgment test across multiple institutions. Am J Pharm Educ.

[16] Gardner AK, Costa P (2024). Predicting surgical resident performance with situational judgment tests. Acad Med.

